# Effective Removal of Different Heavy Metals Ion (Cu, Pb, and Cd) from Aqueous Solutions by Various Molecular Weight and Salt Types of Poly-γ-Glutamic Acid

**DOI:** 10.3390/molecules29051054

**Published:** 2024-02-28

**Authors:** Sheng-Yen Tsai, Chao-Kai Chang, Pei-Yu Wei, Shi-Ying Huang, Mohsen Gavahian, Shella Permatasari Santoso, Chang-Wei Hsieh

**Affiliations:** 1 Department of Food Science and Biotechnology, National Chung Hsing University, South Dist., Taichung City 402202, Taiwan; jeff7780522@gmail.com (S.-Y.T.); kai70219@nchu.edu.tw (C.-K.C.); wei12123@smail.nchu.edu.tw (P.-Y.W.); 2 College of Ocean Food and Biological Engineering, Jimei University, No. 43 Yindou Rd., Xiamen 361021, China; johnhuang@jmu.edu.cn; 3 Department of Food Science, National Pingtung University of Science and Technology, Pingtung 912301, Taiwan; mg@mail.npust.edu.tw; 4 Department of Chemical Engineering, Widya Mandala Surabaya Catholic University, Surabaya 60114, Indonesia; shella@ukwms.ac.id; 5 Department of Chemical Engineering, National Taiwan University of Science and Technology, Daan Dist., Taipei 106221, Taiwan; 6 Department of Medical Research, China Medical University Hospital, Taichung City 404333, Taiwan

**Keywords:** poly-γ-glutamic acid, modified salt, heavy metal, bioflocculation, potentially toxic elements

## Abstract

In light of industrial developments, water pollution by heavy metals as hazardous chemicals has garnered attention. Addressing the urgent need for efficient heavy metal removal from aqueous environments, this study delves into using poly-γ-glutamic acid (γ-PGA) for the bioflocculation of heavy metals. Utilizing γ-PGA variants from *Bacillus subtilis* with different molecular weights and salt forms (Na-bonded and Ca-bonded), the research evaluates their adsorption capacities for copper (Cu), lead (Pb), and cadmium (Cd) ions. It was found that Na-bonded γ-PGA with a high molecular weight showed the highest heavy metal adsorption (92.2–98.3%), particularly at a 0.5% concentration which exhibited the highest adsorption efficiency. Additionally, the study investigated the interaction of γ-PGA in mixed heavy metal environments, and it was discovered that Na-γ-PGA-HM at a 0.5% concentration showed a superior adsorption efficiency for Pb ions (85.4%), highlighting its selectivity as a potential effective biosorbent for wastewater treatment. This research not only enlightens the understanding of γ-PGA’s role in heavy metal remediation but also underscores its potential as a biodegradable and non-toxic alternative for environmental cleanup. The findings pave the way for further exploration into the mechanisms and kinetics of γ-PGA’s adsorption properties.

## 1. Introduction

As industrial development progresses, industries generate substantial amounts of wastewater containing heavy metals, which severely threaten public drinking water’s safety when discharged into rivers [1]. Copper (Cu), lead (Pb), and cadmium (Cd) are prevalent heavy metal elements in industrial wastewater and present severe risks to human health [2]. According to guidelines from the World Health Organization, the maximum permissible limits for Cu, Pb, and Cd in drinking water are 1.0, 0.01, and 0.005 mg/L, respectively [3]. However, the concentrations in industrial discharges often exceed these limits. Excessive intake can cause liver poisoning, nausea, vomiting, diarrhea, muscle cramps, renal failure, sensory disturbances, damage to the central nervous system, decreased growth in children, damage to bone marrow, and even fatalities [4,5]. Hence, mitigating or eradicating the presence of high levels of heavy metals in wastewater, particularly copper (Cu), lead (Pb), and cadmium (Cd), before releasing it into bodies of water, represents a viable approach to addressing this issue [6].

To date, various conventional treatment processes such as ion exchange [7], chemical precipitation [8], chemisorption [9,10], electrodialysis [11], reverse osmosis [12], coagulation and flocculation [13], and flotation [14] have been used to remove heavy metals from water. Moreover, researchers have explored the effects of emerging technologies, such as cold plasma [15] and ohmic heating [16], for the degradation of potentially toxic elements and their effects on heavy metals in water and aqueous solutions. Nevertheless, these methods present notable disadvantages, such as the necessity for substantial quantities of chemicals, persistent solubility issues with metals, elevated capital and operational expenditures, and the production of considerable amounts of sludge [17]. Among the conventional methodologies for extracting heavy metals from wastewater, chemical-based separation techniques are widely implemented across industrial sectors. They are among the most productive and established methods [18]. Nevertheless, this approach may ineffectively eliminate metal ions in low concentrations [19]. 

In recent years, there has been a growing emphasis on biological approaches for treating metal-bearing effluents, with several of these methods progressing toward commercialization. Notably, biosorption has emerged as an up-and-coming alternative to traditional techniques for the removal of metal ions [20]. The removal of heavy metals such as Pb (II) and Hg (II) in wastewater was demonstrated in our previous study utilizing modified cellulose, an organic polymeric substance. This was achieved through the electrostatic interaction between negatively charged modified cellulose and metal cations [6]. Another organic polymeric substance, extracellular polymeric substance (EPS), is synthesized naturally by microorganisms including yeast, bacteria, algae, and fungi. It possesses biodegradable agent properties and functions as a biocoagulant, thereby substantially mitigating the environmental and health risks of chemical flocculants and coagulants [21,22,23]. Bioflocculants/biocoagulants demonstrate excellent heavy metal absorption capabilities, as the presence of ion groups and hydrophobic areas in the polymeric substances enable them to react with heavy metal ions through mechanisms such as charge neutralization, sweep coagulation, bridging, and patch flocculation [24]. After flocculation, the resulting larger agglomerates can be removed or separated by filtration, straining, or flotation [1] and potentially reused as biological fertilizer or for renewable energy production [25,26]. Thus, bioflocculants/biocoagulants are of interest in industry and academia and have the potential to be an alternative to chemical flocculants/coagulants.

Poly-γ-glutamic acid (γ-PGA) is a type of EPS predominantly produced and secreted by various *Bacillus* species through fermentation. It possesses the advantageous characteristics of being water-soluble, biodegradable, consumable, and non-toxic to humans and the environment [27]. Multiple studies have demonstrated the versatile applications of γ-PGA in various fields, such as food science, cosmetics, medicine, and agriculture [28]. The chemical structure of γ-PGA is composed of repeating units of L-glutamic acid, D-glutamic acid, or DL-glutamic acid [29], which are joined by amide connections between α-amino and γ-carboxylic acid groups [27]. Negative charges on the glutamic acid units allow γ-PGA to be soluble in water and provide it with favorable biocompatibility [30]. In addition to that, γ-PGA has different ionic forms and molecular masses ranging from 10,000 to 2 million Da, depending on the fermentation process [31]. The diverse conformational states, hydrogen bonding capabilities, and polyanionic characteristics of γ-PGA endow this biopolymer with a versatile and multifunctional profile, which is conducive to a wide range of beneficial biological functionalities [32]. 

**Figure 1:**
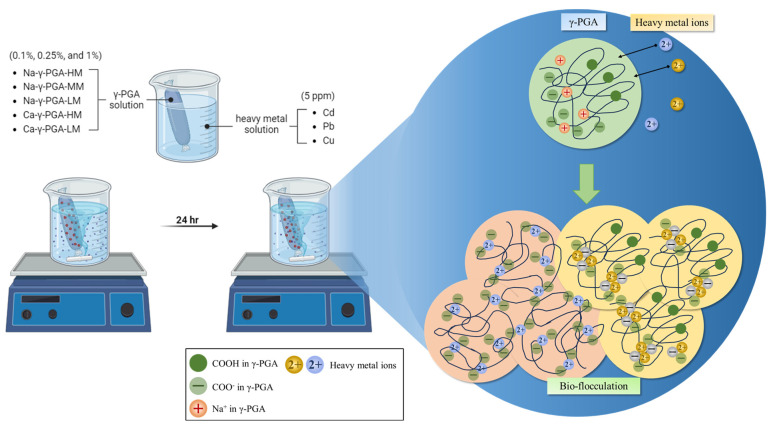
The heavy metal adsorption treatment and γ-PGA bioflocculation schematic diagram.

Extensive research has demonstrated the potential of γ-PGA as a heavy metal biosorption agent [33]. Liu, Li [34] conducted a study on the utilization of γ-PGA derived from Bacillus pumilus to eliminate heavy metals from sewage. The study successfully achieved significant removal rates for various heavy metals including Zn, Cr, V, Cd, Pb, and Ni. Mu, Zhang [35] studied the adsorption properties of γ-PGA and its complexes with gelatin and Fe_3_O_4_ on heavy metal ions. They observed significant adsorption capabilities for Cu^2+^, Pb^2+^, and Cd^2+^. In addition to that, a previous study by Wang, Liu [36] demonstrated that γ-PGA can form complexes with heavy metal ions through ion exchange, thereby facilitating the flocculation process of these ions (Figure 1). In this study, γ-PGA (H-form) was employed for the flocculation of Pb^2+^ and Cu^2+^, revealing that the biosorption ability was affected by the secondary structure and the quantity of COOH and COO^−^ groups. Hence, γ-PGA’s capacity as a biosorption agent has significant promise for effectively eliminating heavy metal pollutants in waste, especially in wastewater.

Further research conducted by Sakamoto and Kawase demonstrated that the adsorption capacity of γ-PGA on heavy metals can be influenced by the presence of salts such as Na in γ-PGA [37] because of the competing occupancy of active sites on the γ-PGANa surface by Na^+^ and ion metals [38]. It is also crucial to consider the molecular weight of γ-PGA, as prior investigations have demonstrated that distinct molecular weights (400–2500 kDA) exhibit varying capacities for heavy metal removal in water [39]. Nevertheless, the impact on different salt types and molecular weights of γ-PGA remains unclear. Therefore, this study aim to investigate the chelation effect between Cu, Pb, and Cd and γ-PGA with varying molecular weights and salt types (Na-bonding and Ca-bonding) in a simulated environment reflective of industrial wastewater treatment processes.

## 2. Results and Discussion

### 2.1. Different Types of γ-PGA Adsorption Effects on Heavy Metal Ions

**Figure 2:**
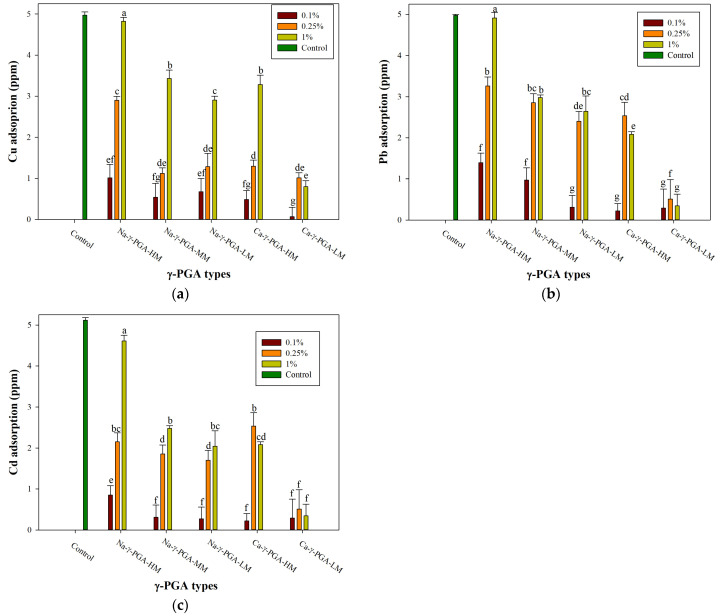
Different salts modified γ-PGA and concentrations adsorption to heavy metals: (**a**) Cu, (**b**) Pb, (**c**) Cd adsorption. ^a–g^ Means followed by different superscripts with different modified γ-PGA and concentrations are significantly different at *p* < 0.05. Control: Without γ-PGA dissolved.

This process effectively facilitates the adsorption of heavy metals. Our examination focused on various types of γ-PGA, specifically Na-γ-PGA (HM, MM, and LM) and Ca-γ-PGA (HM, LM), at concentrations of 0.1%, 0.25%, and 1%. The adsorption was observed over 24 h using 5 ppm heavy metal standard solutions (Cu, Pb, and Cd) for extracellular analysis. As depicted in Figure 2a–c, the results indicate the distinctive adsorption efficiencies of different γ-PGA types for Cu, Pb, and Cd.

This study demonstrated that heavy metals can pass through the dialysis membrane and chelate with γ-PGA, thereby forming large agglomerates. The adsorption of γ-PGA induces a charge gradient between the polymer solution and the metal ion solutions, enabling the heavy metals to traverse the dialysis membrane until the adsorption reaches saturation. Figure 2 demonstrates that under identical molecular weight conditions, the Na-form of γ-PGA exhibited a superior adsorption effectiveness for Cu, Pb, and Cd compared to the Ca-form. Figure 2a–c shows that the Cu, Pb, and Cd adsorption rate of Na-γ-PGA-HM at an identical concentration of 1% was found to be 1.47, 2.36, and 2.21 times higher than that of Ca-γ-PGA-HM, while Na-γ-PGA-LM achieved an adsorption rate 3.71, 7.79, and 5.95 times greater than Ca-γ-PGA-LM. These findings have a similar result to previous research. The research conducted by Inbaraj, Wang [40] focusing on the impact of light metal ion concentration on mercury adsorption has revealed significant insights. As the study increased the concentration of light metal ions from 5 mg/L to 80 mg/L, a notable decrease in mercury adsorption was observed. This decrease was particularly evident, with no adsorption occurring at concentrations of 50 mg/L for calcium ions and 80 mg/L for sodium or potassium ions. The study highlighted a pronounced decline in mercury adsorption in the presence of varying concentrations of calcium, sodium, and potassium ions—at levels of 5, 10, 20, and 30 mg/L—with calcium showing a two-fold reduction in adsorption efficiency compared to sodium and potassium ions. The marked influence of Ca^2+^ on heavy metal binding can be rationalized through the hard and soft acids and bases (HSAB) principle proposed by Pearson [41]. According to this principle, Ca^2+^, a hard acid, should more readily bind with hard bases such as the carboxyl groups on γ-PGA. This finding is in contrast to Cu, Pb, and Cd, which are categorized as soft acids and exhibit different binding characteristics. 

Figure 2a–c further elucidate the influence of different molecular weights of γ-PGA on the adsorption of various heavy metal ions. It is evident that γ-PGA exhibits a greater adsorption capacity with a higher molecular weight than its lower-molecular-weight counterparts. For instance, at an identical concentration of 1%, the adsorption rates of Cu, Pb, and Cd by Na-γ-PGA-HM are 1.63, 1.86, and 2.26 times higher, respectively, than those by Na-γ-PGA-LM. Similarly, Ca-γ-PGA-HM demonstrates adsorption rates of 4.12, 6.07, and 6.06 times greater than Ca-γ-PGA-LM. This phenomenon may be attributed to the secondary structure of the γ-PGA molecular chain. Prior studies have indicated that the average molecular weight of γ-PGA influences the formation of a helical structure, enhancing cationic ion adsorption [38,42]. The results overwhelmingly suggest that Na-γ-PGA-HM possesses superior adsorption capabilities.

The data presented in Figure 2 elucidate the significant influence of γ-PGA concentration on its capacity to adsorb heavy metals. Specifically, when the concentration of γ-PGA was elevated from 0.1% to 1%, a marked increase in the adsorption capacity for Cu, Pb, and Cd was observed across different molecular weight variants of Na-form γ-PGA. The adsorption capacity for Cu increased by factors of 4.75, 6.34, and 4.30 for Na-γ-PGA-HM, Na-γ-PGA-MM, and Na-γ-PGA-LM, respectively. For Pb, the increases were identical to those observed for Cu, whereas for Cd, the factors were 3.53, 3.07, and 8.78, respectively, indicating a particularly pronounced increase for the low-molecular-weight variant. These findings suggest that lower concentrations of γ-PGA result in decreased heavy metal adsorption capacities, which can be attributed to a scarcity of available chelating groups. This limitation hinders the formation of stable coordinate bonds within the ligand’s ring structure, essential for effective adsorption. Conversely, at a 1% concentration of γ-PGA, the abundance of chelating groups facilitates the establishment of numerous coordinate bonds with heavy metal ions, significantly enhancing the adsorption process. This enhancement in adsorption capacity at higher γ-PGA concentrations is corroborated by the observed stability in the composition of the ligand ring, which plays a critical role in the efficient sequestration of heavy metals [31]. However, Ca-form γ-PGA shows different behavior; it is observed that a 0.25% concentration of Ca-γ-PGA-LM exhibits a higher adsorption capacity for Cu, Pb, and Cd ions compared to a 1% concentration. Similarly, Ca-γ-PGA-HM at 0.25% also shows enhanced removal efficiency for Pb and Cd ions. Previous studies have reported that the Ca-form of γ-PGA exhibits different molecular conformations in aqueous solutions, resulting in distinct physical characteristics compared to its Na-form [43]. Additionally, it has been shown to possess a higher flocculating activity, especially at high concentrations [44], underscoring the importance of ionic forms in determining the functional characteristics of γ-PGA. Sakamoto and Kawase [38] have reported that the adsorption capacity decreased with increasing biosorbent dosage, implying a reduction in biosorption efficiency. The increase in adsorbent dosage caused an increase in available adsorption sites, resulting in an increase in the removal amount and simultaneously an increase in diffusion path length due to the agglomeration of the adsorbents, resulting in a decrease in the removal amount. The reduction in adsorption capacities with increasing adsorbent dosage was also attributed to their aggregation or agglomeration, causing a suppression of the increase in adsorption sites [45]. The research underscores the importance of γ-PGA concentration in optimizing the adsorption efficiency of heavy metals, highlighting the potential for adjusting γ-PGA salt types, molecular weight, and concentrations in environmental remediation strategies to achieve maximal heavy metal uptake.

### 2.2. γ-PGA Dose−Heavy Metal Adsorption Activity Relationship 

**Figure 3:**
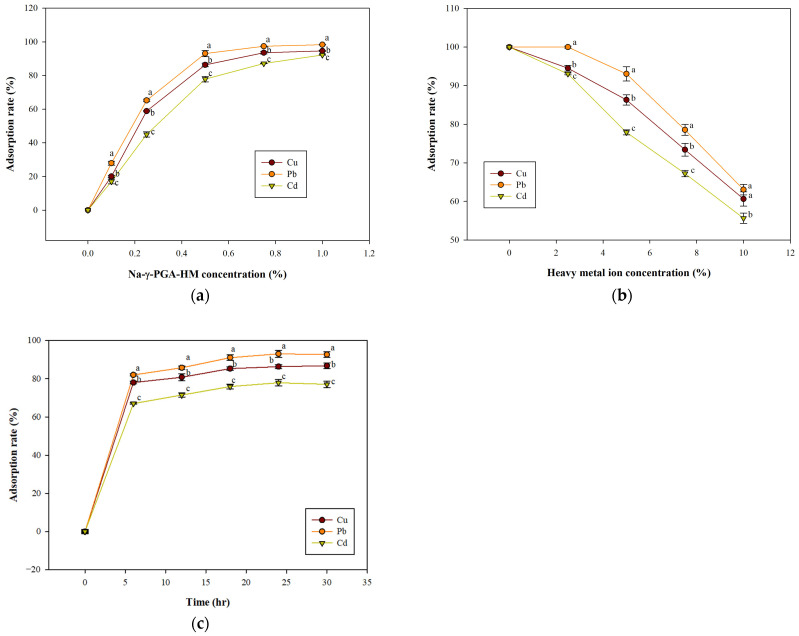
Assessing the impact of varying concentrations of Na-γ-PGA-HM (**a**), heavy metal ions (**b**), and the time during dialysis (**c**) on the adsorption of heavy metals. ^a–c^ Means followed by different superscripts in the same concentrations are significantly different at *p* < 0.05.

Figure 3 illustrates the dose–response curves for Na-γ-PGA-HM in Cu, Pb, and Cd adsorption. Under equivalent doses of Na-γ-PGA-HM, Pb exhibited the highest rate of removal, followed by Cu, with Cd showing the lowest removal percentage. As the dose of Na-γ-PGA-HM was incrementally increased from 0.01% to 1% for lead concentrations at 5 ppm, the removal percentages for Cu, Pb, and Cd increased significantly. Specifically, the Cu, Pb, and Cd removal percentages increased to 94.62%, 98.31%, and 92.22%, respectively. However, it was noted that the amount of metal adsorbed per unit weight of γ-PGA decreased with increasing doses of γ-PGA. A concentration of 0.5% Na-γ-PGA-HM demonstrated the highest efficiency. This finding suggests that the metal binding effectiveness of γ-PGA is not directly proportional to its higher doses. In a previous study, Sakamoto and Kawase [38] utilized γ-PGA for cesium removal and dye sorption. Such a phenomenon could be ascribed to the diminished binding capacity utilization of γ-PGA at elevated concentrations, potentially due to its aggregation or agglomeration, which could reduce the accessibility of binding sites [31,45]. Furthermore, it is important to consider that at high doses of γ-PGA, particle interactions may lead to the desorption of weakly bound metal ions from the γ-PGA surface [45]. This phenomenon suggests that the binding efficiency of γ-PGA is more pronounced at lower concentrations. Consequently, employing smaller doses of γ-PGA could be more effective for treating and removing heavy metals. 

The relationship between the concentration of heavy metal ions and their adsorption efficiency onto sodium γ-polyglutamic acid with Na-γ-PGA-HM is underscored by the findings illustrated in Figure 3b. The data reveal a noticeable decrease in the adsorption rates of Cu, Pb, and Cd ions as their concentrations diminish, indicating a declining trend in adsorption efficiency. Specifically, the adsorption rates for Cu, Pb, and Cd ions fell from 94.56% to 60.62%, 100% to 63.04%, and 93.06% to 55.63%, respectively. This trend is consistent with prior studies, which postulate that at lower metal ion concentrations, ion exchange mechanisms primarily facilitated by carboxyl groups become the dominant process for metal ion adsorption. In contrast, at elevated concentrations of metal ions, chemical interactions involving the amide groups of Na-γ-PGA-HM may play a more significant role [40,45,46]. Additionally, the rate constants for metal ion adsorption decrease as the concentration of the metal ions increases. Notably, lead ions are adsorbed more rapidly than cadmium ions under varying concentrations, which may be attributed to the distinct mechanisms that govern their interaction with Na-γ-PGA-HM. This differential adsorption rate highlights the variances in binding capacities and the specific chemical interactions that occur between the metal ions and the functional groups present in Na-γ-PGA-HM [36]. 

Figure 3c presents data on the adsorption rates observed during the dialysis process, highlighting the swift adsorption capabilities of sodium γ-polyglutamic acid with a high molecular weight (Na-γ-PGA-HM). The process reached completion within a 16 h timeframe, with Pb ions demonstrating the highest rate of adsorption, whereas Cd ions displayed the lowest. This differential in adsorption rates is further compounded by the observed correlation between increased metal ion concentration and decreased rate constants, suggesting that Pb ions are adsorbed more rapidly than Cd ions under similar conditions. The variation in adsorption rates between Pb and Cd ions can be explained by the underlying mechanisms that govern their interaction with Na-γ-PGA-HM [47]. As previously discussed, these mechanisms include ion exchange processes and chemical interactions that are influenced by the specific properties of the metal ions, such as their electronegativities and tendencies to form complexes. The preferential adsorption of Pb ions over Cd ions may be due to a more favorable interaction between Pb ions and the functional groups present in Na-γ-PGA-HM [48].

The findings delineated in Figure 3 shed light on the differential adsorption dynamics exhibited by γ-PGA towards various metal ions, with Pb ions demonstrating notably higher adsorption rates compared to both Cu and Cd ions. Specifically, at a γ-PGA concentration of 0.5%, the adsorption rates for Pb ions were observed to be 1.07 and 1.19 times greater than those for Cu and Cd ions, respectively. This pattern of adsorption behavior is consistent with previous research, which suggests that the biosorption capacity of γ-PGA is significantly influenced by its coordination mode and conformation changes upon metal ion complexation [48]. A critical aspect of this observation is the role of structural modifications in γ-PGA that occur upon complexation with Pb^2+^ ions. These modifications are believed to enhance the polymer’s ability to engage in binding interactions through its carboxyl (COOH) groups [31]. In contrast, the structure of γ-PGA remains largely unchanged after complexation with Cu^2+^ ions, which appears to limit its capacity for similar binding interactions [36]. This distinction highlights the intricate relationship between the molecular structure of γ-PGA and the specific characteristics of metal ions, which together determine the efficacy of γ-PGA’s biosorption capabilities. 

### 2.3. Adsorption Efficiency of γ-PGA in Analog Mixed Environment 

The conducted experiment revealed that a 0.5% concentration of sodium γ-polyglutamic acid with a high molecular weight (Na-γ-PGA-HM) was the most effective in removing heavy metals from an aqueous solution. However, it is essential to acknowledge that this finding was specific to a particular environmental context of heavy metal adsorption. The adsorption process involves the formation of complexes between heavy metal ions and γ-PGA through ion exchange mechanisms. The effectiveness of this process is influenced by the differing electronegativities of the heavy metal ions, which affect the strength and nature of the ion exchange interactions [36].

**Figure 4:**
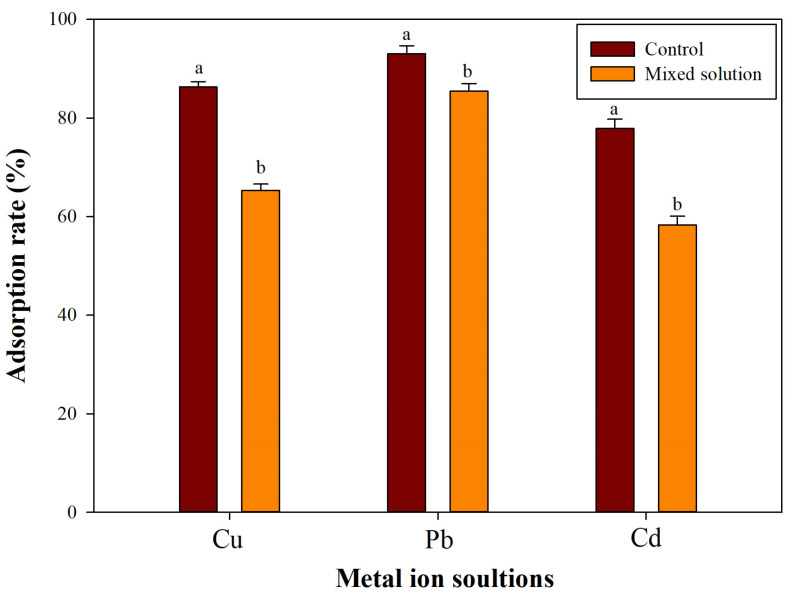
Na-γ-PGA-HM adsorption of the mixed heavy metals solution. ^a,b^ Means followed by different superscripts in the same Na-γ-PGA-HM concentrations are significantly different at *p* < 0.05. Control: singular heavy metal ion.

Given the variability in electronegativities among different heavy metal ions, it is imperative to conduct further research to determine if Na-γ-PGA-HM exhibits comparable adsorption effectiveness in environments containing multiple metal contaminants. In Figure 4, it was observed that in scenarios with mixed heavy metal ions, there was a notable decrease in the adsorption efficiency for Cu, Pb, and Cd, with reductions of 21.10%, 7.61%, and 19.53%, respectively. This indicates a competitive adsorption environment where the presence of multiple metal ions affects the binding efficiency of γ-PGA to each type of metal ion. Interestingly, γ-PGA showed a preferential selectivity for Pb over Cd, which suggests that Pb^2+^-γ-PGA complexes are more stable compared to those formed with Cd. This selectivity could be attributed to the higher electronegativity of copper ions, which interferes with the binding of lead and cadmium ions due to copper’s ability to form more labile hydrolyzed species similar to lead, which are more readily bound by γ-PGA [31]. Furthermore, among the studied metal ions, Pb^2+^ uniquely influences the conformation of γ-PGA. This can be attributed to two main reasons: the higher electronegativity of Pb^2+^, which enhances its affinity towards the carboxylate (COO-) groups of γ-PGA [31], and its tendency to form coordination complexes with a larger number of water molecules compared to other ions [49]. The latter results in a larger hydrated radius, potentially disrupting hydrogen bonds within the γ-PGA molecule and leading to significant conformational changes [48,50]. These findings underscore the complexity of the adsorption process in multi-metal environments and highlight the need for comprehensive studies to better understand the interactions between γ-PGA and mixed heavy metal ions. Such insights are crucial for optimizing the use of γ-PGA in environmental remediation strategies, especially in contexts where multiple types of heavy metal pollutants are present.

## 3. Materials and Methods

### 3.1. Materials

This study utilized five variants of γ-PGA, all produced from *Bacillus subtilis*, supplied by VEDAN Enterprise Corporation (Taichung, Taiwan). These variants differed in molecular weight: high molecular weight (HM, MW > 1500 kDa), middle molecular weight (MM, MW > 1000 kDa), and low molecular weight (LM, MW > 400 kDa); and differed in salt types (Na^+^-bonded and Ca^2+^-bonded), resulting in the following samples: Na-γ-PGA-HM, Na-γ-PGA-MM, Na-γ-PGA-LM, Ca-γ-PGA-HM, and Ca-γ-PGA-LM. Stock metal solutions, used as atomic absorption standards at 1000 μg/mL, included copper (Cu(NO_3_)_2_), lead (Pb(NO_3_)_2_), and cadmium (Cd(NO_3_)_2_) (Avantor Performance Materials Taiwan Co. Ltd., Zhubei City, Taiwan). All other reagents and chemicals were procured from Sigma-Aldrich (St. Louis, MO, USA).

### 3.2. Biosorption of Heavy Metals to γ-PGA 

The adsorption method is according to a previous study with small modifications [36]. Five different types of γ-PGA were solubilized in 50 mL of deionized water and subsequently enclosed within dialysis membranes with a molecular weight cut-off of 14 kDa (MEMBRA-CEL^®^, VISKASE^®^ Companies, Inc., Darien, IL, USA); the dialysis membranes’ surface area was set at 80 cm^3^. These membranes, laden with γ-PGA, were immersed in 450 mL of an aqueous solution infused with heavy metals to achieve a final concentration of 5 ppm for the heavy metals. The concentrations of the γ-PGA solutions were prepared to be 0.1%, 0.25%, and 1% (*w*/*v*), respectively. The selection of γ-PGA concentrations for the dialysis procedure included 0.1%, 0.25%, and 1%. The adsorption process was designed to mimic industrial wastewater treatment protocols [18,19,51,52]. Dialysis was performed for 24 h at a meticulously controlled temperature (25 °C) and pH value (pH = 7) (Figure 1). Additionally, 2 mL extracted from the exterior solution was collected for UV spectroscopy analysis (190–260 nm) to verify the absence of leaked components in the exterior solution. Control experiments, conducted without incorporating γ-PGA, demonstrated minimal heavy metal adsorption by the dialysis membrane or within the stirred cell during the ultrafiltration procedure. 

### 3.3. Analysis of Heavy Metal Adsorption to γ-PGA 

The mechanism underlying the removal of heavy metal ions is attributed to the precipitation/coagulation facilitated by γ-PGA. To elucidate their contributions to the removal of heavy metals, our approach involved separating the precipitated/coagulated matter from the solution via the dialysis membrane. Samples extracted from the exterior solution of the γ-PGA dialysis membrane underwent analysis to determine the presence of Cu, Pb, and Cd. This analysis was conducted using flame atomic absorption spectrometry (Z-5000, Hitachi, Ltd., Tokyo, Japan). The spectrometry air–acetylene flame was used as an atomization source, and hollow cathode lamps (Hitachi, Ltd., Tokyo, Japan) for each measured element as a light source. The quantification was based on measuring the absorption at characteristic wavelengths specific to each element. Each sample was analyzed in triplicate, and the average concentrations of the heavy metals were determined based on the readings obtained from the instrument [53]. 

The maximum absorbance values of the samples were assessed using a calibration curve established with heavy metal concentrations (mg/L). The determination of metal concentration (*N*) in water samples is conducted by the following formula [46]:

(1) N mgL=A×V1V

where *A* was obtained from the calibration curve of the heavy metal concentration (mg/L), *V* was the original volume (mL) of water sample, and *V*_1_ was the final volume (mL) of the pre-treated water sample.

### 3.4. Analog Mixed Environment Assay

Three heavy metals (Cu, Pb, and Cd) at a concentration of 5 ppm were introduced into a mixture of various heavy metal adsorption modules. Following a 24 h adsorption period, the efficacy of γ-PGA in adsorbing these heavy metals was assessed. This evaluation focused on the potential multiple adsorption effects occurring within the assay of the mixed heavy metals solution [54].

### 3.5. Statistical Analysis

In this study, all experiments were measured in three replicates. All statistical analyses were conducted using SAS 21 (SAS Institute Inc., Cary, NC, USA). Statistical analysis was performed with a one-way analysis of variance (ANOVA) and Duncan’s multiple range tests. Statistical significance was considered at *p* < 0.05.

## 4. Conclusions

This research provides compelling evidence that the concentration and molecular weight of γ-PGA are critical determinants in its ability to remove heavy metals from aqueous solutions. A significant finding from the recent research highlights the superior performance of Na-forms of γ-PGA with a high molecular weight (Na-γ-PGA-HM) compared to Ca-γ-PGA-HM in the context of heavy metal removal. This comparison reveals that Na-γ-PGA-HM exhibits removal efficiencies for various heavy metals that span from 92.22% to 98.31%. These results underscore the enhanced effectiveness of γ-PGA in heavy metal adsorption when utilized at higher concentrations and molecular weights. Furthermore, the study identified that a concentration of 0.5% Na-γ-PGA-HM is optimally effective in adsorbing heavy metals at a concentration of 5 ppm. In the context of mixed heavy metal solutions, simulating wastewater environments with Cu, Pb, and Cd, Na-γ-PGA-HM displayed a particularly high adsorption capacity for Pb. These findings highlight the exceptional potential of γ-PGA as an efficient biosorbent for the remediation of heavy-metal-contaminated wastewater. Future research efforts are directed towards a deeper understanding of the adsorption mechanisms and kinetics of γ-PGA in heavy metal removal. Additionally, further studies aim to explore the adsorptive selectivity of γ-PGA towards various heavy metals in more detail. This ongoing research will contribute to the development of more effective environmental cleanup strategies, leveraging the natural adsorption capabilities of γ-PGA to mitigate the impact of heavy metal pollution.

## Data Availability

Data are contained within the article.
